# MAPPING SIDEWALK FALL RISKS USING BIG DATA AND MACHINE LEARNING

**DOI:** 10.1093/geroni/igac059.1104

**Published:** 2022-12-20

**Authors:** Fabio Miranda, Maryam Hosseini

**Affiliations:** University of Illinois at Chicago, Chicago, Illinois, United States; New York University, Brooklyn, New York, United States

## Abstract

Outdoor falls are a leading cause of fatal and non-fatal injuries in the US. These falls are more likely to occur due to environmental hazards, inclement weather, unsafe construction zones, or poor sidewalk condition. Fall prevention programs targeted at older adults must therefore be informed by the interplay between weather condition, urban development, and built environment. Current practices, however, are limited by the lack of comprehensive data describing the condition of pedestrian facilities at fine, human scale, limiting the effectiveness of these programs. To address these shortcomings, we propose a multi-pronged approach leveraging urban data and machine learning techniques to create city-scale inventories describing sidewalk features that can inform safe strategies and programs for community mobility. We will cover the creation of multiple data sets, including computing shadow / shade from building geometries, detection of sidewalk surface material from street-level images, and creation of sidewalk networks from satellite images.

